# Enamel Wear of Antagonist Tooth Caused by Dental Ceramics: Systematic Review and Meta-Analysis

**DOI:** 10.3390/jcm11216547

**Published:** 2022-11-04

**Authors:** Manuel León Velastegui, José María Montiel-Company, Rubén Agustín-Panadero, Carla Fons-Badal, María Fernanda Solá-Ruíz

**Affiliations:** 1 School of Dentistry, Faculty of Health Sciences, Universidad Nacional de Chimborazo, Riobamba 060103, Ecuador; 2 Department of Stomatology, Faculty of Medicine and Dentistry, University of Valencia, 46010 Valencia, Spain

**Keywords:** tooth wear, enamel wear, dental porcelain, ceramic, antagonist

## Abstract

Background: This systematic review and meta-analysis aimed to evaluate the wear of the antagonist tooth in ceramic restorations. Material and methods: This study was carried out based on Preferred Reporting Items for Systematic Review and Meta-analysis (PRISMA) recommendations; it was also registered in PROSPERO (register number: CRD42022316252). Three databases were consulted in the literature search, Embase, Scopus, and Web of Science. The citation searching was conducted by two researchers independently. The clinical studies that evaluated wear in antagonist teeth concerning ceramic restoration were included. Twelve articles were selected after eliminating duplicates ones and applying the inclusion criteria, and two were chosen through citation. Fourteen articles were considered for the qualitative and quantitative analysis (meta-regression and meta-analysis). Results: The mean linear wear of the antagonist tooth in relation to feldspathic was 8.914 μm, for lithium disilicate it was 0.018 μm, and for zirconia it was 0.257 μm. The mean volumetric wear of the antagonist tooth in relation to feldspathic was 0.273 mm^3^, for hybrid ceramic it was 0.030 mm^3^, for lithium disilicate it was 0.018 mm^3^, and for zirconia it was 0.014 mm^3^. The mean natural tooth wear was 0.7974 μm per month. Tooth wear caused by zirconia at six months was 31.755 μm, at 12 months it was 24.648 μm, and at 24 months it was 20.662 μm. Conclusions: Feldspathic produces greater wear of the antagonist tooth from ceramic restorations linearly and volumetrically. In addition, zirconia generates the least wear that will decrease over time, and it will be equal to or less than the natural wear in the tooth.

## 1. Introduction

Loss of occlusal substance from a tooth that is not caused by caries [1], especially by antagonizing opposing surfaces resulting in wear [2], occurs on the hard enamel and dentin surfaces of teeth, physiologically or pathologically [3]. It occurs between tooth and tooth or between the tooth and the restoration that is in contact with. This process could be accelerated by introducing materials whose properties differ from the tooth structure: toughness, resistance to fracture, surface roughness, or greater hardness than the tooth [4]. That is why these characteristics were associated with dental wear, which is multifactorial, including processes such as abrasion, attrition, and corrosion. To recover lost dental tissue, ceramics are used as the first-choice restorative material, with various materials and classifications that help us with relevant information for their use [5]. 

In clinical practice, it is easy to observe the wear of natural teeth antagonistic to prostheses made with ceramics that have been in service for many years. Different patterns and depths of wear in the natural tooth can be observed, questioning why this type of wear occurs in certain patients and not in others. In these terms, there are a series of factors that cannot be controlled, so we should at least thoroughly know the wear properties of the material used in order to avoid tooth structure loss in clinical situations [4,6]. 

## 2. Materials and Methods

The systematic literature review was carried out in accordance with the PRISMA recommendations (PRISMA: Preferred Reporting Items for Systematic Reviews and Meta-Analyses) [7] with prior registration in PROSPERO (Registration number CRD42022316252). 

The PICO question (population, intervention, comparison, outcome) was: What restorative material causes greater wear on the opposing natural tooth? The designations used were “P” (patient): patients who have a fixed prosthesis; “I” (intervention): bridges, crowns, or overlays of different types of ceramics (lithium disilicate, feldspathic, zirconia, etc.); “C” (comparison): healthy tooth; and “O” (outcome): tooth wear. The terms in each section were defined respectively. The said terms were subjected to Boolean operations set as “OR” and “AND”. An electronic search took place in the following databases: Embase, Scopus, Web of Science (WOS): (TS = (“dental ceramic”) OR TS = (“dental porcelain”) OR TS = (“lithium disilicate”) OR TS = (“metal ceramic”) OR TS = (“feldspathic”) OR TS = (“alumina”) OR TS = (“Zirconia”) OR TS = (“glass ceramic”)) AND (TS = (“crown”) OR TS = (“onlay”) OR TS = (“bridge”) OR TS = (“overlay”)) AND (TS = (“wear”) OR TS = (“tooth wear”) OR TS = (“enamel wear”) OR TS = (“occlusal wear”) OR TS = (“antagonist wear”) OR TS = (“dental wear”)) AND (TS = (“in vivo”) OR TS = (“clinical trial”) OR TS = (“randomized”)).

The systematic review and meta-analysis spanned all the literature published up to 30 June 2022. The following inclusion criteria were applied: clinical trials, in humans or in vivo; adult patients with fixed partial dentures with occlusal coverage; healthy teeth antagonists; and studies that measured wear numerically. No restriction was placed on the language of publication. The exclusion criteria included: patients with bruxism, primary teeth, implant prosthetics, and in vitro studies. 

Two members of the research team (M.L.V., M.F.S.-R.) carried out duplicated database searches independently. The headings and abstracts were selected by applying inclusion and exclusion criteria. The first researcher (M.L.V.) also collected data for relevant variables and carried out the systematic review. After that, the meta-analysis was performed by a third researcher who was not involved in the selection process (J.M.M.-C.). 

The variables registered were author, year of publication, title, journal, type of study, sample size (*n* = patients), gender, inclusion and exclusion criteria, intervention (*n* = crowns), tooth position or region, material of the restoration, material composition, manufacturer, patient follow-up time, wear measurement methods, software used for measurements, wear measurements, lineal or volumetric wear, and quality of the studies. 

**Figure 1:**
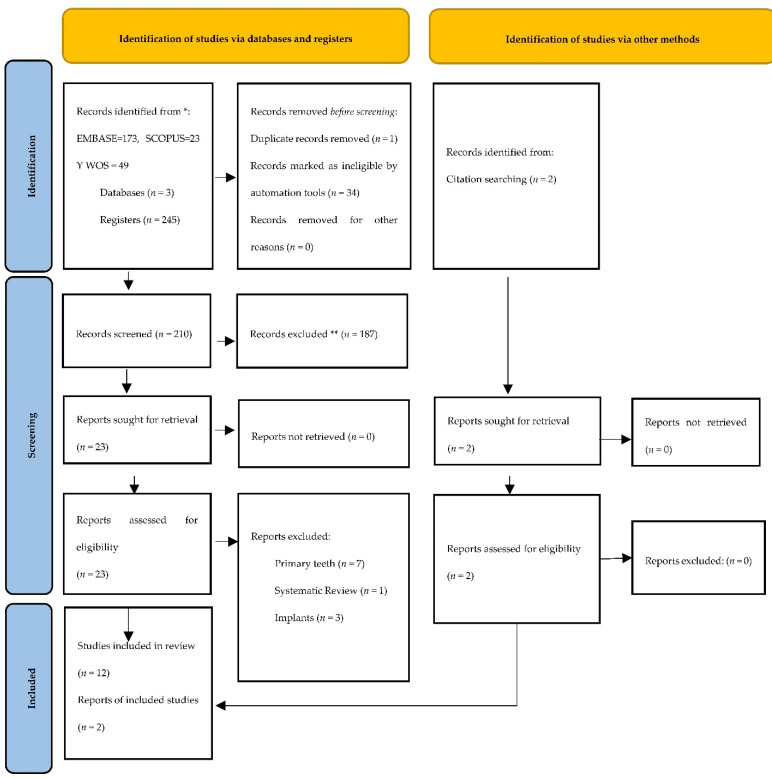
Preferred Reporting Items for Systematic Reviews and Meta-Analyses (PRISMA) flow diagram. * Consider, if feasible to do so, reporting the number of records identified from each database or register searched (rather than the total number across all databases/registers). ** If automation tools were used, indicate how many records were excluded by a human and how many were excluded by automation tools.

The researchers independently analyzed the quality of the studies. The quality assessment results are presented in Figure 1. Clinical trials were identified, and Cochrane’s RoB 2.0 tool was used for quality assessment.

This study measured the amount of wear of the dental structure produced by the different materials in the antagonists, as well as the natural or physiological wear produced between two natural teeth in the same patient at different times, considering their standard deviations. The standardized difference was used as the effect measure by combining the included studies with a random-effects model using the Mantel–Haenszel method through meta-regression. A pooled meta-analysis was performed to analyze zirconia wear over time in a random-effects model, and the mean difference was calculated as a measure of effect. Heterogeneity was assessed using the Q test, *p*-value, and I^2^, considering the existence of heterogeneity when the *p*-value of the Q test is less than 0.1 and the I^2^ greater than 50%, indicating moderate heterogeneity.

The software used for the meta-regression and meta-analysis was Comprehensive Methanalyses 3.0. 

## 3. Results

The initial electronic search identified 173 studies in Embase, 23 in Scopus, and 49 in WOS. Out of the 245 articles, 240 remained after removing duplicates; 187 studies were excluded after reviewing titles and abstracts. A total of 23 articles were eligible for full-text reading, and, subsequently, 11 articles were eliminated for not meeting the inclusion criteria (type of restoration and permanent teeth). Finally, 12 studies were chosen, and another 2 studies were included by manual search. This means that 14 studies were used for qualitative and quantitative analysis (Figure 1).

**Figure 2:**
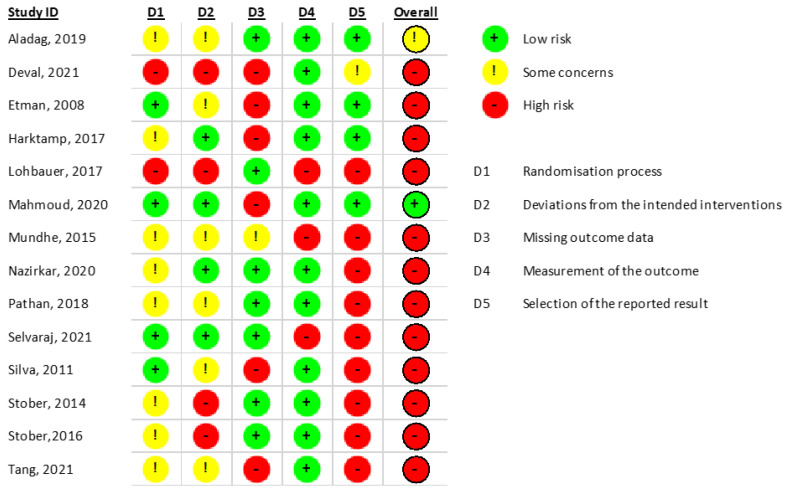
Quality of the studies in Cochrane’s RoB 2.0 for clinical trials [4,9,10,11,12,13,14,15,16,17,18,19,20,21].

Fourteen clinical trials were identified using Cochrane’s RoB 2.0 tool for the quality assessment [8], obtaining one study with a low risk of bias [9], another one with an unclear risk of bias [4], and twelve studies with a high risk of bias (Figure 2).

Qualitative and quantitative analysis included 14 articles. The samples varied between 9 and 60 patients; patient ages ranged from 18 to 73 years; the studies considered premolars and molars, maxillary and mandible teeth, and the buccal region; follow-up times ranged from 3 to 36 months; there were different wear evaluation methods; and Geomagic and Polyworks software were the most used (Table 1). 

The ceramics used in the studies were feldspathic, zirconia, and lithium disilicate. The wear of the tooth was analyzed volumetrically and linearly (Table 2). 

### 3.1. Tooth Wear—Linear Analysis

The mean wear was obtained from the included studies in the meta-regression that analyzed the linear wear of the antagonist tooth caused by ceramic. These results were presented in descending order according to the type of ceramic (feldspathic: 8.9149 μm, lithium disilicate: 0.0189 μm, and zirconia: 0.2574 μm). 

**Figure 3:**
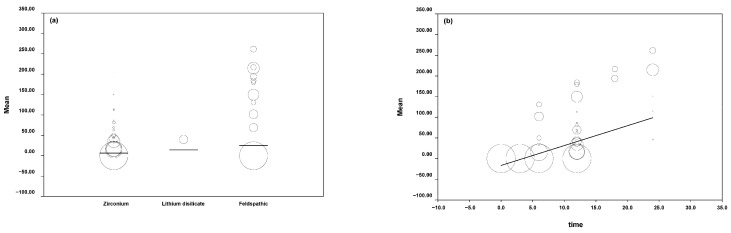
(**a**) Graphic of the linear wear produced in the opposing tooth. (**b**) Linear wear with respect to time.

The model was obtained with a Q test = 46,607.28 (*p* = 0.0000); this is indicative that the variable time is significant in the model. The beta coefficient of time was 4.8086 with a *p*-value = 0.000 and CI at 95% (4.7616–4.8556) (Figure 3).

### 3.2. Tooth Wear—Volumetric Analysis

The means of volumetric wear produced in the dental structure according to the antagonist ceramic material were feldspathic: 0.2734 mm^3^, lithium disilicate: 0.0189 mm^3^, hybrid ceramic: 0.0305 mm^3^, and zirconia: 0.0141 mm^3^.

**Figure 4:**
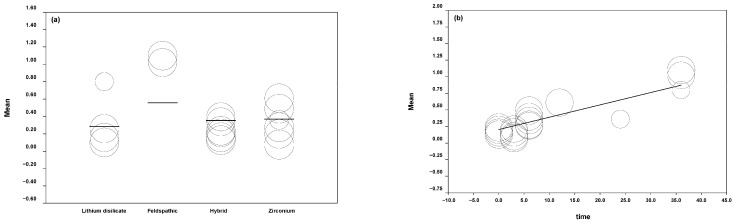
(**a**) Graphic of the volumetric wear in antagonist tooth. (**b**) Volumetric wear concerning time.

The model was obtained with a Q test = 75.08 (*p* = 0.0000); this is indicative that the variable time is significant in the model, giving a predictive capacity of 83% (R^2^ = 8.3). The beta coefficient of time was 0.0186 with a *p* value = 0.0003 and CI at 95% (0.0085–0.0287) (Figure 4).

### 3.3. Tooth Wear Control Group o Natural Tooth

**Figure 5:**
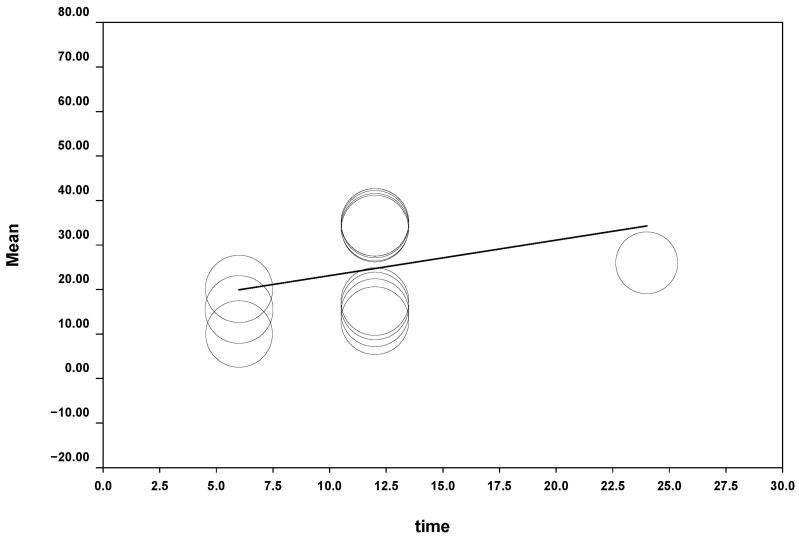
Natural wear of antagonist tooth.

The wear of the antagonist tooth of the control group could be calculated when carrying out the meta-regression using the control group measurements shown in several studies. The wear was 0.7974 μm per unit of time analyzed, with a *p*-value of 0.1522 and CI at 95% (−0.2941–1.889). This wear would produce 9.5658 μm of approximate wear in one year between natural teeth. The model was obtained with a Q test = 2.05 (*p*-value = 0.1522) (Figure 5).

### 3.4. Tooth Wear—Zirconia Analysis

**Figure 6:**
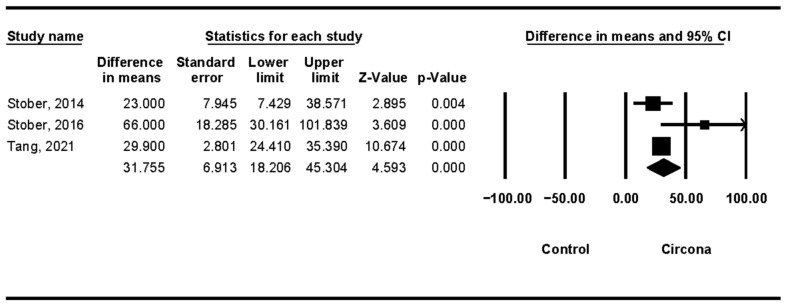
Zirconia linear wear (6 months).

The linear wear of the zirconia group was estimated with the random-effects model. Two studies that analyzed the linear wear [19,20,21], were combined and compared with their control group over six months, with a mean difference of 31.755 μm obtained. In the meta-analysis, there was moderate heterogeneity between the combined studies. (Q test = 4.652; *p*-value = 0.098) (Figure 6).

**Figure 7:**
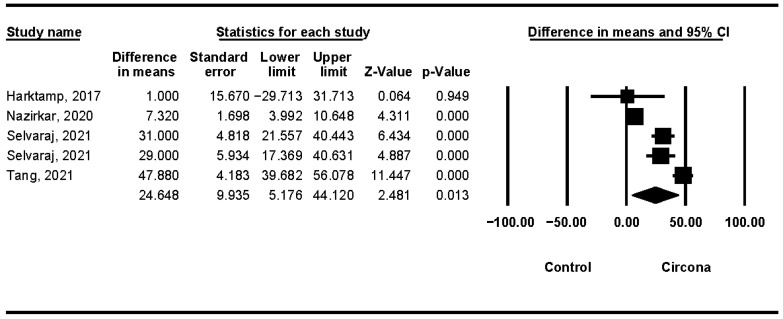
Zirconia linear wear (12 months).

In 12 months, a mean difference of 24.648 μm was obtained. In the meta-analysis, there was a high degree of heterogeneity between the combined studies [12,15,17,21] (Q test = 99.518; *p*-value = 0.0000) (Figure 7).

**Figure 8:**
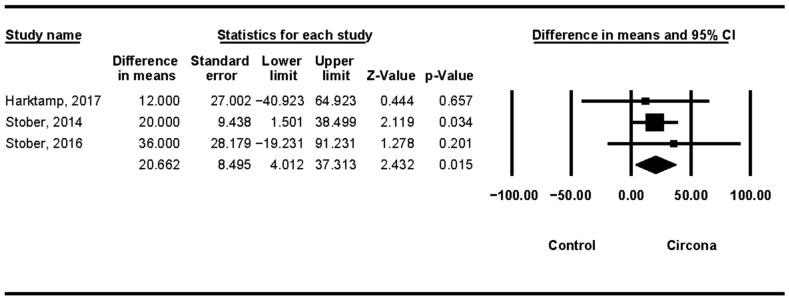
Zirconia linear wear (24 months).

In 24 months, a mean difference of 20.662 μm was obtained in the meta-analysis [12,19,20], (Q test = 0.404; *p*-value = 0.817) (Figure 8).

## 4. Discussion

The wear of the occlusal tooth structure antagonistic to other surfaces is usually linked to abnormal mechanical processes other than chewing, such as attrition and abrasion [21], and is associated with the hardness of restorative materials. With the development of technology and the appearance of new materials, it is necessary to know the wear that these ceramics produce on natural teeth since they are used in daily practice, and many others do not have clinical studies that justify their use in the different treatments in patients.

The ceramics are very varied in the studies found. Zirconia was used in the most significant number of investigations since this material is the hardest one regarding association with wear. A range of zirconia has been studied, such as Lava (3M ESPE) by [12,13,14,17], Zenostar (Wieland dental) by [19,20,21], and other studies by [4,9,15,16]. Feldspathic was also used as a coating material for different nuclei as a metallic alloy in [14,18,22], as ceramic infrastructures in studies by [11,18], and covering zirconia in the study by [9]. Lithium disilicate is present in a smaller number of studies [4,11,15,18], as well as the hybrid ceramics that are only present in the study by [4], since there is so much variety of ceramics and compositions. Ceramics become complex to associate or classify, as explained in [23].

There is no protocol or a straightforward way to quantify the wear; even the measurement methods vary. The model or the initial and final images to measure the difference between them vary from study to study [24]. However, the use of the intraoral scanner is currently being opted for, which helps to obtain the samples directly from a three-dimensional image [25]. Some researchers [4,12,21] used to apply a spray before obtaining the image, which could lead to distortion in the image [26], as well as data loss when transferring the captured images to STL format [27].

Most studies make impressions with the addition of silicones or polyvinylsiloxanes, and then the images are captured by profilometers [11,13,22], or extraoral scanners are used later, as in the studies by [14,15,16,17,18,19,20]. This could lead to distortion, since the image should previously go through the printing process and obtain the model. However, both are accepted and allow the obtainment of a clear starting image [28,29]. The methodology by [16] is the only one that differs from all since it measures wear by the means of pixelated images obtained through the microscope.

Different software brands calculated the difference between the initial and final images; the most frequently used were the Geomagic Qualify and Polywork. They allow the obtainment of the difference in wear, getting very varied results. However, we have at our disposal new methods and software that evaluate surface wear according to current needs.

The wear of antagonist teeth can be influenced by the average values of masticatory forces and the position of teeth. This varies from individual to individual and is also influenced by food consistency [30,31,32,33]

In terms of linear wear, based on the meta-regression carried out by [9,16,18], it was found that feldspathic is the ceramic that produces the most significant wear [9,11,14]. We can observe greater wear values for feldspathic used as a comparison group to other ceramics. Lithium disilicate causes intermediate wear [20], which means that the average wear produced is less than the comparison with zirconia. The material that produces the least linear wear in the dental tissue is zirconia, which has been used in most studies carried out by [9,16]. Zirconia also maintains lower values than feldspathic and lithium disilicate, which is why it was used as a moderator in the meta-regression. This result contrasts with the study performed by [15], in which the greatest wear was produced by zirconia when compared to lithium disilicate, coinciding with the volumetric wear in our study.

Regarding wear by volume analyzed in the studies by [4,13,18,21], the greatest wear is produced by feldspathic, which coincides with the result obtained in linear wear by [18,34]. Feldspathic has always been considered the comparison material and gold standard for most investigations [35], but it produces the greatest wear both linearly and volumetrically in vivo and in vitro [36]. Lithium disilicate is used in meta-regression as a moderator, being the most stable. Two studies by [4,18] coincide with [37], which found that it produces greater wear than zirconia. 

Hybrid ceramics were only analyzed by [4], who compared Cerasmart and Vita Enamic with zirconia and lithium disilicate. The first ceramics have a more stable and lower behavior than the other ones, especially Cerasmart, as reported by [38], who mentions that the wear of this type of restoration material occurs because of the separation of the filling materials from the matrix due to its use, producing greater wear that increases its surface roughness. So, it is recommended to polish this type of restoration periodically; finally, zirconia shows the least wear, coinciding with linear wear.

However, the natural tooth also presents physiological wear. According to the study by [39], it is an average of 29 μm in molars and 15 μm in premolars in one year, which is opposed to the amount of natural wear found in the study by [16] with values of 15.5 μm at six months and 16.3 μm at 12 months (without specifying whether they are premolars or molars). In this meta-regression, the average wear is 0.7974 μm, and per unit of time analyzed is 9.56 μm. The wear would occur in one year between natural teeth without specifying the premolar or molar tooth. 

This meta-regression determined that zirconia is the material that shows less wear on the opposing tooth. However, when performing a comparison of means in a meta-analysis, a decrease was found in the wear produced at the points or contact surfaces that were initially related to the antagonist, as stated by [21]. The comparison of glazed or polished contact surfaces was a variable analyzed in several studies, especially those that used zirconia, obtaining results of less wear when it is polished [13,14,15,17,21,40], unlike the higher values when it is glazed [16,19,20], and what was mentioned before has been verified in the reviews carried out by [34].

Within the limitations of this review, we find the variability of ceramic materials on the market that are not necessarily clinically tested before being marketed. The different methods of wear measuring could also cause discrepancies between the studies, and the different software used for image analysis would cause variability in the data obtained. The polishing or glazing of the surfaces, as well as surface roughness, should be associated as variables for the analysis of the wear of the dental structure.

The wear of the opposing tooth produced by ceramics should be analyzed in clinical studies using materials according to the type, composition, use, and preparation, or clearer variables that help us in our clinical decisions, carried out with a longer follow-up time to know if the wear is maintained or declines with use, for which the methodology should use efficient and currently standard tools, which would allow, in the future, the creation of similar studies that would allow more reliable and comparable measurements to be obtained.

## 5. Conclusions

Feldspathic produces greater wear in the antagonist tooth in ceramic restorations, linearly and volumetrically. In addition, zirconia generates the least wear that will decrease over time, and it will be equal to or less than the natural wear in the tooth.

## Figures and Tables

**Table 1:** Follow-up, method, and software employed for wear measurements.

Author (Year)	Sample (*n*)	Age	Tooth or Buccal Region	Follow-Up Time	Wear Evaluation Method	Software
Aladag, 2019 [4]	24	18–50	*	3 and 6 months	Intraoral scanner Bluecam (Cerec 3D)	David-Laserscanner, V3.10.4, Berlin, Germany
Deval, 2019 [10]	30	18–40	First molar, maxillary or mandibular	1 year	Polyvinyl siloxane impression, cast poured in type IV gypsum and scanned using 3D white light scanner (SmartSCAN 3D-HE, Breuckmann, Heiligenhaus, Germany)	Polyworks InnovMetric software
Etman, 2008 [11]	48	20–60	First premolar: 6 (5 = maxilar, 1 mandibular), second premolar: 18 (11 = maxilar, 7 mandibula), first molar: 52 (22 = maxilar, 30 = mandibula), second molar: 14 (5 = maxilar, 9 = mandibula)	6, 12, 18, and 24 months	Polyvinyl siloxane impression scanned with a noncontacting laser profilometer (Keyence LC-2400 series laser displacement meter)	Scan-Surf Sotware
Hartkamp, 2017 [12]	9	*	Premolar and molar maxilar and mandibular teeth	12 and 24 months	Intraoral scanner LAVA C.O.S (3M ESPE, Seefeld, Germany)	Geomagic Qualify 2012 1.2, 64 bit version; Geomagic Inc., Morrisville, NC, USA)
Lohbauer, 2017 [13]	10	Mean: 45.2 (11.9)	First premolar to molar region.	24 months	Polyvinyl siloxane impressions, replicas were manufactured from the epoxy resin material and scanned using scanning electron microscopy (Leitz ISI SR 50, Akashi, Japan)	Surfer 9 (Golden software, Golden, CO, USA)
Mahmoud, 2020 [9]	34	20–60	Premolar and molar teeth	3, 6, and 12 months	Polyvinyl siloxane impressions and replicas were manufactured from the epoxy resin cast and photographed using USB digital microscope with a built-in camera connected with an IBM-compatible personal computer using a fixed magnification.	WSxM software
Mundhe, 2015 [14]	10	18–35	Premolar and molar teeth	12 months	Polyvinyl siloxane impressions were poured in type III gypsum and scanned using a Smart Scan 3d HD scanner (Breukmann).	Polyworks InnovMetric software
Nazirkar, 2020 [15]	30	20–40	14 maxillary and 16 mandibular molars	12 months	Polyvinyl siloxane impression and scanned using ZirkonZannSagoo (Arti Germany)	Polyworks InnovMetric software
Pathan, 2018 [16]	60	*	Premolar and molar teeth	6 and 12 months	Polyvinyl siloxane impressions were poured and the casts were scanned using a 3D laser scanner (REXCAN CS = Solutionix, Seoul, Korea).	Geomagic Qualify (3D Systems, Inc., Morrisville, NC, USA)
Selvaraj, 2021 [17]	14	18–45	Premolar and molar teeth	12 months	Polyvinyl siloxane impressions were scanned with a SmartSCAN 3D HE Scanner (Breuckmann)	Polyworks InnovMetric software
Silva, 2011 [18]	31	24–62	*	6, 12 months	Polivinyl siloxane impressions were scanned with a 3D scanner (es1 Scanner, Etkon, Gräfelfing, Germany)	SAS PROC MIXED, SAS Institute, Cary, NC, USA
Stober, 2014 [19]	20	21–73	Molar teeth	12 months	Polyvinyl siloxane impressions and poured in type IV gypsum and digitalized with laser scanner 3D and profilometer (Laserscan 3D)	MATCH 3D, version 1.6
Stober, 2016 [20]	20	21–73	Molar teeth	12, 24, and 36 months	Polyvinyl siloxane impressions and poured in type IV gypsum and digitalized with laser scanner 3D and profilometer (Laserscan 3D)	MATCH 3D, version 1.6
Tang, 2021 [21]	43	21–71	43 teeth: 16 maxillary first molar, 3 maxillary second molar, 1 maxillary second premolar, 19 mandibular first molar, 3 mandibular second molar, and 1 mandibular second premolar.	6 months	Introral scanner (InEos X5 3D scanner, Dentsply Sirona Inc., Berlin, Germany)	Geomagic control 2014.3.0 software (Geomagic Co. Ltd., Morrisville, NC, USA)

* Not specified.

**Table 2:** Material and wear analyzed.

Author (Year)	Material	Wear
Aladag, 2019 [4]	IPS e.max CAD (EM) (lithium disilicate)	Volumetric
	Cerasmart (GC) (resin matrix ceramic)	
	Suprinity (VS) (zirconia-reinforced lithium silicate) (ZLS)	
	Enamic (VE) (resin matrix ceramic (polymer-infiltrated-ceramic-network (PICN))	
Deval, 2019 [10]	Metal ceramic Monolithic zirconia	Linear
Etman, 2008 [11]	Procera coping AllCeram Simidur S2 alloy Experimental glass ceramic	Linear
Hartkamp, 2017 [12]	Lava Plus	Linear
Lohbauer, 2017 [13]	Lava Plus	Linear/Volumetric
Mahmoud, 2020 [9]	Feldspalthic veneering VM9 katana blanks	Linear
Mundhe, 2015 [14]	Y-TZP Lava Bellabond Plus and Ceramco 3	Linear
Nazirkar, 2020 [15]	Zirconia yttrium tetragonal Ingots-IPS e. Max	Linear
Pathan, 2018 [16]	Monolithic Zirconia	Linear
Selvaraj, 2021 [17]	Lava (Polished Dialite ZR, Brasseler) Lava (Glazed, IPS ivocolor glaze paste, Ivoclar Vivadent)	Linear
Silva, 2011 [18]	Argedent 62, Argen, San Diego, and IPS d.SIGN veneer, Ivoclar Vivadent IPS Empress 2 core ceramic with IPS Eris for E2 veneering ceramic, Ivoclar Vivadent IPS e.max Press core ceramic without a veneering ceramic	Volumetric
Stober, 2014 [19]	Zenostar Zr Translucent	Linear
Stober, 2016 [20]	Zenostar Zr Translucent	Linear
Tang, 2021 [21]	Zenostar Zr Translucent	Linear/Volumetric
